# The Double Burdens of Mental Health Among AIDS Patients With Fully Successful Immune Restoration: A Cross-Sectional Study of Anxiety and Depression in China

**DOI:** 10.3389/fpsyt.2018.00384

**Published:** 2018-08-24

**Authors:** Xiaojie Huang, Kathrine Meyers, Xinchao Liu, Xia Li, Tong Zhang, Wei Xia, Jiahua Hou, Aixin Song, Haolan He, Chongxi Li, Shenghua He, Weiping Cai, Huolin Zhong, Chengyu Huang, Shuiqing Liu, Hui Wang, Xuemei Ling, Ping Ma, Rongxia Ye, Gang Xiao, Taisheng Li, Ding Ding, Kristine Yaffe, Hui Chen, Yaokai Chen, Hao Wu

**Affiliations:** ^1^Center for Infectious Diseases, Beijing You'an Hospital, Capital Medical University, Beijing, China; ^2^The Aaron Diamond AIDS Research Center, The Rockefeller University, New York, NY, United States; ^3^Infectious Diseases Department, Peking Union Medical College Hospital, Beijing, China; ^4^Infectious Diseases Department, Yunnan AIDS Care Center, Kunming, China; ^5^Institute of Infectious Diseases, The Eighth People's Hospital of Guangzhou, Guangzhou, China; ^6^Infectious Diseases Department, The Third People's Hospital of Kunming, Kunming, China; ^7^Department of Infectious Diseases, Chongqing Infectious Disease Medical Center, Chongqing, China; ^8^Department of Infectious Diseases, Guiyang Public Health Clinical Center, Guiyang, China; ^9^Department of Clinical AIDS Research, The Third People's Hospital of Shenzhen, Shenzhen, China; ^10^Department of Hematology, The Third People's Hospital of Hengyang, Hengyang, China; ^11^Department of Infectious Disease, The Second Affiliated Hospital of Medical School of the Southeast University, Tianjin, China; ^12^Department of Infectious Diseases, The Sixth People's Hospital of Hangzhou, Hangzhou, China; ^13^Department of Infectious Diseases, The First Hospital of Changsha, Changsha, China; ^14^Shanghai Public Health Clinical Center, Shanghai, China; ^15^Department of Psychiatric and Neurology and Department of Epidemiology and Statistics, University of California, San Francisco, San Francisco, CA, United States; ^16^School of Biomedical Engineering, Capital Medical University, Beijing, China

**Keywords:** HIV-1, anxiety, depression, Hospital Anxiety and Depression (HAD) scale, mental health, prevalence, risk factors

## Abstract

**Background:** Anxiety and depression continue to be significant comorbidities for people with HIV infection. We investigated the prevalence of and factors associated with anxiety and depression among adult HIV-infected patients across China.

**Methods:** In this cross-sectional study, we described clinical and psychosocial variables related to depression and anxiety in 4103 HIV-infected persons. Doctors assessed anxiety and depression by asking patients whether they had experienced anxiety or depression in the prior month. Patients also self-administered the Hospital Anxiety and Depression (HAD) scale; those with score ≥8 on HAD-A/D were considered to be at high risk of anxiety or depression.

**Results:** Associations between socio-demographic, psychosocial, and ART-related clinical factors and risk of depression or anxiety were investigated using multivariable logistic regression. Among patients assessed between 9/2014 and 11/2015, 27.4% had symptoms of anxiety, 32.9% had symptoms of depression, and 19.0% had both. Recentness of HIV diagnoses (*P* = 0.046) was associated with elevated odds of anxiety. Older age (*P* = 0.004), higher educational attainment (*P* < 0.001), employment (*P* = 0.001), support from family / friends (*P* < 0.001), and sleep disturbance (*P* < 0.001), and number of ART regimen switches (*P* = 0.046) were associated with risk of depression, while neither sex nor transmission route showed any associations. There were no significant associations with HIV-specific clinical factors including current CD4^+^ T cell count and current viral load.

**Conclusions:** Prevalence of symptoms of anxiety and depression is high in this cohort of treatment-experienced HIV patients. Psychological and social-demographic factors, rather than HIV disease status, were associated with risk of depression and anxiety. This finding highlights the need to deliver interventions to address the mental health issues affecting HIV-infected persons with fully successful immune restoration across China.

## Introduction

Over half of all HIV-1-infected individuals suffer from mental health disorders (1–3) and anxiety and depression are common comorbidities in HIV-infected populations (4–6). The impact of mental health issues on HIV patients in resource-limited settings is often underestimated due to a lack of education and awareness both among physicians and patients, resulting in insufficient and *ad-hoc* screening and diagnosis of mental health conditions in this population (4, 7). The pathophysiology of anxiety/depression among HIV-infected patients is unclear, but may be related to clinical factors (e.g., the ability of HIV to infect the central nervous system (CNS) or impact of antiretroviral medications) or psychosocial in nature (e.g., availability of social support, issues related to substance abuse) (8–10). Recent studies have largely focused on the impact of specific antiretroviral agents (e.g., efavirenz) on mental health (11, 12). Regardless of its etiology, anxiety and depression is clinically important in this population and has the potential to impact quality of life, adherence to anti-retroviral medications, cognition, cause sleep disturbance and may weaken patients' immune system (13–16).

There are currently no guidelines to manage psychiatric disorders in the HIV clinic setting in China, despite the reality that these disorders adversely affect the course of HIV infection (17). Major anxiety and depression frequently go unrecognized and untreated and may foster severe consequences, such as rapid disease progression, suicide, high-risk behaviors and immune system impairment (18–20). In this study, we characterized the prevalence of symptoms of anxiety and depression in Chinese HIV-infected patients, identified risk factors, and sought to assess clinical impacts of anxiety and/or depression on treatment outcomes. We also investigated the utility of the Hospital Anxiety and Depression (HAD) instrument to identify missed-diagnoses when compared to a clinician-initiated one-item question asking patients to self-report anxiety and depression in the previous month.

## Methods

We conducted a cross-sectional study among 4724 HIV-infected adults on treatment, collecting data from patients at 20 HIV treatment clinics across China. All participants provided written informed consent to complete a survey and have their medical data abstracted from their medical records. The study was approved by the Beijing You'an Hospital institutional review board.

Each participant completed the Hospital Anxiety and Depression (HAD) scale questionnaire, which consists of seven items each relating to depression (HAD-D) and anxiety (HAD-A) respectively. High risk of anxiety and depression was defined as a HAD score ≥8 on each subscale (21). Before self-administration of the HAD, clinicians asked patients if they had experienced any depressed mood or anxiety in the previous month and results were coded yes vs. no.

Demographic, behavioral, and psychosocial data were collected through self-administered survey. Clinical data was abstracted from medical records. This included HIV-specific data, including date of HIV diagnosis, history of medical conditions, current CD4^+^ cell counts and HIV RNA levels, and antiretroviral therapy (ART) regimen. The Pittsburgh Sleep Quality Index (PSQI) questionnaire, a 19-item questionnaire that assesses seven sleep components, was completed by each participant to evaluate sleep disturbance during the prior month. We used the PSQI cut off of a global score >5 on the PSQI instrument (15) (sensitivity of 90% and specificity of 87%) to define sleep disturbance in this cohort (22).

## Statistical analysis

Descriptive statistics are presented as means with standard deviations (SDs), or counts with proportions, as appropriate. Two sample *t*-tests were used to compare means, and χ^2^-tests were used to compare proportions. Logistic regression analysis was used to investigate associations between demographic, behavioral, psychosocial, and clinical factors and anxiety or depression. Odds ratios (ORs) for depression or anxiety were estimated with 95% confidence intervals (CIs). Factors with a *P*-value < 0.10 in univariate Logistic models were initially included in the multivariate Logistic model and were then eliminated using backward selection. Crude ORs are reported from univariate analysis; adjusted ORs are reported from multivariable logistic regression. All *P*-values were 2-sided, and *P*-values < 0.05 were considered significant. Analyses were conducted using SPSS 21.0.

## Results

### Characteristics of study population

4103 HIV-infected persons were eligible for data anlaysis with the mean age being 37.6 ± 11.7 years (Table 1). Over three-quarters of the cohort were male patients. The cohort represented a diverse sample of educational achievement and employment status. Over 40% were infected through anal sex. The median time from HIV diagnosis to study enrollment was 27 months (interquartile range [IQR], 11–58 months); median duration of ART was 18 months (IQR, 6–43 months); 15.5% had a current CD4^+^ T cell count below 200 cells/mm^3^, and 40.8% were virally-suppressed. Three-quarters of patients were on first-line ART regimen (Table 1). Over 60% had disclosed their HIV status to their family members and close to 70% expressed feeling supported by their family members. In contrast, just over one quarter had disclosed their status to a friend.

**Table 1:** Demographic, clinical, and psychosocial characteristics of 4103 HIV patients.

**Demographic factors**		**Clinical factors**		**Psychosocial factors**	
**Age, years**		**Current CD4 cell count, cells/mm^3^**		**Family history of mental illness**	
<30	1,175 (28.5)	<50	128 (3.1)	Yes	85 (2.1)
30–50	2,306 (56.2)	50–199	506 (12.3)	No	4,004 (97.6)
>50	569 (13.9)	200–499	2,160 (52.6)	Missing	14 (0.3)
Missing	53 (1.3)	≥500	1,272 (31.0)	Personal history of mental illness	
Gender		Missing	37 (0.9)	Yes	65 (1.6)
Male	3,204 (78.1)	Current viral load		No	4,026 (98.1)
Female	831 (20.3)	Undetectable	1,672 (40.8)	Missing	12 (0.3)
Missing	68 (1.7)	Detectable	1,781(43.4)	Current sleep medication use	
Education		Missing	650 (15.8)	Yes	236 (5.8)
< High school	1,528 (37.2)	Time since HIV diagnosis		No	3,867 (94.2)
High school	988 (24.1)	<3 months	297 (7.2)	Current antidepressant use	
> High school	1,527 (37.2)	≥3 months	374 (91.3)	Yes	197 (4.8)
Missing	60 (1.5)	Missing	58 (1.4)	No	3,884 (94.7)
Transmission route		Adherence to ART		Missing	22 (0.5)
Heterosexual	1,589 (38.9)	≥95	3,642 (88.8)	HIV status disclosed to friends	
Homosexual/bisexual	1,730 (42.2)	<95	461 (11.2)	Yes	1,092 (26.6)
Blood transfusion	39 (1.0)	Current EFV-based regimen		No	2,996(73.0)
PWID	216 (5.3)	Yes	2,944 (71.8)	Missing	15 (0.4)
Unknown	520 (12.7)	No	1,159 (28.2)	HIV status disclosed to family	
Marital status		Duration of EFV exposure		Yes	2,464 (60.1)
Married	1,698 (41.4)	0 month	1,159 (28.2)	No	1,628 (38.7)
Divorced	456 (11.1)	<3 months	681 (16.6)	Missing	11 (0.3)
Widowed	132 (3.2)	≥3 months	2,217 (54.0)	Support from family/friends	
Single	1,718 (41.9)	Missing	46 (1.1)	Yes	2,803 (68.3)
Missing	99 (2.4)	Duration of ART		No	1,239 (30.2)
Employment		<1 year	1,514 (36.9)	Missing	61 (1.5)
White collar	1,158 (28.2)	≥1 year	2,528 (61.6)		
Blue collar	1,835 (44.7)	Missing	61 (1.5)		
Unemployed	280 (6.8)				
Student	830 (20.2)				

### Prevalence of depression and anxiety

1,349 (32.9%) and 1,125 (27.4%) HIV-infected persons had symptoms of depression and anxiety based on the HAD-D and HAD-A, respectively, among whom 779 persons (19.0%) had symptoms consistent with both depression and anxiety. Out of 1,344 patients whose HAD-D score indicated likely depression, 715 (53.2%) did not report feeling depressed in the previous month in response to the clinician-initiated question (Table 2). Meanwhile, 578 (21.0%) of those whose HAD-D score suggested that they were not depressed responded that they had in fact experienced depressed mood in the last month.

**Table 2:** Agreement of depression diagnosis based on HAD-D and self-report [n (%)].

**Patient report of depression**	**Depression diagnosis based on HAD-D**≥**8**		**Total**
	**Presence**	**Absence**	
Presence	629 (46.8)	578 (21.0)	1,207
Absence	715 (53.2)	2,169 (79.0)	2,884
Total	1,344	2,747	4,091

### Factors associated with depression and anxiety

In univariate analyses, factors associated with likely depression (HAD-D ≥ 8) or anxiety (HAD-A ≥ 8) included age, sex, education, transmission route, employment, support from family/friends, time of diagnosed with HIV infection, drug adherence, duration of EFV exposure, duration of ART, number of ART regimens, and sleep disturbance. There were no significant associations with HIV-specific clinical factors including current CD4^+^ T cell count and current viral load.

In the final multivariate model, factors associated with depression based on the HAD included age (*P* = 0.004), education (*P* < 0.001), and employment (*P* = 0.001), while factors associated with anxiety included time since HIV diagnosis (*P* = 0.046), number of ART regimen (*P* = 0.046), employment (*P* < 0.001), support from family/friends (*P* < 0.001), and sleep disturbance (*P* < 0.001) (Table 3).

**Table 3:** Demographic and clinical factors associated with depression and anxiety.

**Factors**	**Depression (HAD-D**≥**8)**			**Anxiety (HAD-A**≥**8)**		
	**% of subjects**	**Crude OR (95% CI)**	**Adjusted OR (95% CI)**	**% of subjects**	**Crude OR (95% CI)**	**Adjusted OR (95% CI)**
**DEMOGRAPHIC AND PSYCHOSOCIAL FACTORS**						
**Age, years** ‡**&**						
<30	26.3%	1	1	26.5%	1.17 (0.93–1.48)	
30–50	35.6%	1.55 (1.33–1.81)	**1.35 (1.13–1.62**)	28.9%	1.32 (1.07–1.64)	
>50	34.6%	1.48 (1.20–1.84)	**1.29 (1.00–1.68)**	23.6%	1	
**Gender #**						
Female	37.5%	1.31 (1.12–1.54)		29.6%	1.15 (0.97–1.36)	
Male	31.5%	1		26.7%	1	
**Education** ‡						
< High school	39.9%	1.96 (1.68–2.29)	**1.89 (1.54–2.31)**	27.4%	1.04 (0.88–1.22)	
High school	33.8%	1.51 (1.27–1.80)	**1.45 (1.19–1.78)**	29.0%	1.13 (0.94–1.35)	
> High school	25.3%	1	1	26.7%	1	
**Transmission route #&**						
Heterosexual	34.7%	1		25.7%	1	
Homosexual/bisexual	28.8%	0.76 (0.66–0.88)		27.7%	1.11 (0.95–1.30)	
Blood transfusion	35.9%	1.06 (0.54–2.05)		30.8%	1.29 (0.65–2.57)	
PWID	48.6%	1.78 (1.34–2.37)		35.6%	1.61 (1.19–2.17)	
Unknown	34.0%	0.97 (0.79–1.20)		28.1%	1.13(0.91–1.41)	
**Marital status**						
Married	35.3%	1		26.7%	1	
Divorced	37.7%	1.11 (0.90–1.38)		31.6%	1.27 (1.01–1.59)	
Widowed	35.6%	1.01 (0.70–1.47)		31.1%	1.24 (0.84–1.82)	
Single	28.6%	0.74 (0.64–0.85)		26.8%	1.01 (0.87–1.17)	
**Employment** ‡**$**						
White collar	25.9%	1	1	24.9%	1	1
Blue collar	33.5%	1.44 (1.22–1.69)	1.05 (0.86–1.28)	27.0%	1.12 (0.95–1.32)	1.17 (0.97–1.41)
Unemployed	29.3%	1.18 (0.89–1.58)	1.02 (0.72–1.44)	23.2%	0.91 (0.67–1.24)	0.93 (0.67–1.31)
Student	42.5%	2.12 (1.75–2.56)	**1.49 (1.18–1.87)**	33.3%	1.51(1.24–1.83)	**1.57 (1.26–1.95**)
**Support from family/friends** ‡**$**						
Yes	29.1%	1	1	24.4%	1	1
No	41.2%	1.71 (1.48–1.96)	**1.80 (1.54–2.10)**	33.7%	1.57 (1.36–1.82)	**1.60 (1.36–1.87)**
**CLINICAL FACTORS**						
**Current CD**_4_ **count, cells/mm**^3^						
<50	32.8%	1		22.7%	1	
50–199	35.0%	1.10 (0.73–1.66)		29.4%	1.43 (0.90–2.25)	
200–499	33.5%	1.03 (0.71–1.51)		28.2%	1.34 (0.88–2.05)	
≥500	31.3%	0.93 (0.63–1.37)		25.9%	1.19 (0.77–1.84)	
**Current viral load**						
Undetectable	34.7%	1.11 (0.97–1.28)		27.8%	1.06 (0.91–1.23)	
Detectable	32.4%	1		26.7%	1	
**Time since HIV diagnosis $**						
<3 months	34.0%	1.05 (0.82–1.35)		34.3%	1.42 (1.10–1.82)	**1.33 (1.00–1.76)**
≥3 months	32.9%	1		26.9%	1	1
**Adherence to ART #&**						
≥95%	32.0%	1		26.7%	1	
<95%	39.5%	1.38 (1.13–1.69)		33.4%	1.38 (1.12–1.70)	
**Duration of EFV exposure #**						
0 month	37.0%	1		27.1%	1	
<3 months	31.9%	0.79 (0.64–0.98)		30.5%	1.18 (0.95–1.47)	
≥3months	31.2%	0.77 (0.67–0.90)		26.7%	0.98 (0.84–1.15)	
**Duration of ART #**						
<1 year	30.2%	1		29.0%	1	
≥1 year	34.8%	1.23 (1.08–1.41)		26.6%	1.13 (0.98–1.30)	
**Number of ART Regimens #$**						
<3	32.6%	1		27.2%	1	1
≥3	45.5%	1.72 (1.09–2.71)		37.7%	1.62 (1.01–2.57)	**1.71 (1.01–2.90)**
**Sleep disturbance** ‡**$**						
Yes	46.5%	3.01 (2.62–3.46)	**3.21 (2.77–3.72)**	43.6%	4.22 (3.63–4.91)	**4.10 (3.51–4.78)**
No	22.4%	1	1	15.5%	1	1

‡ *P < 0.05 in both univariate analysis and multivariate analysis for depression*.

# *P < 0.05 in univariate analysis for depression*.

$ *P < 0.05 in both univariate analysis and multivariate analysis for anxiety*.

& *P < 0.05 in univariate analysis for anxiet*.

*HAD, hospital anxiety and depression; OR, odds ratio; CI, confidence interval; ART, antiretroviral therapy*.

*The bold values are adjusted OR and 95% CI after controlling several confounders*.

## Discussion

This study represents one of the largest epidemiologic studies of the prevalence of symptoms of anxiety and depression among HIV-infected persons in the ART era as measured by the validated and frequently-used HAD scale (22–25). Our estimates of 32.9% prevalence of depressive symptoms and 27.4% prevalence of anxiety symptoms are lower than those reported in a systematic review of studies measuring mental health burden among people living with HIV in China that reported median prevalence of depression and anxiety of 60.4 and 43.1% respectively (2). However the authors of that review caution that their data may not be generalizable due to low quality of studies and high risk of bias in the samples (2) as many of these studies were conducted among former plasma donors and injecting drug users who were infected in the first waves of the HIV epidemic in China and may not be representative of more recently diagnosed individuals who tend to be infected through sexual contact (2). In addition, our study was conducted among patients currently accessing ART whereas earlier studies included both treated and untreated patients. A recent study of current depression among recently-diagnosed men who have sex with men (MSM) found 36% prevalence of depression, (26) in line with our finding of 34% depression prevalence among recently-diagnosed patients. People who inject drugs had the highest prevalence of depression and anxiety, in line with a rich literature documenting the comorbidities of mental health and substance use (4, 27, 28).

Students had higher rates of both anxiety and depression, an effect that remained even after controlling for age. As infections among young people and students continue to rise in China (29), it will become increasingly important to develop mental health interventions that have been shown to be effective for young people in other contexts, including ones that could be group-based, web-based, dyadic or individual (30, 31). Age older than 30 and less education were also associated with increased odds of depression in this cohort, a trend that has been seen in other studies as well (32).

In our study the length of time since HIV diagnosis was found to be associated with high anxiety symptoms, with a significant association between shorter duration since diagnosis and anxiety. Anxiety can be considered a normal emotional response to the reality of living with HIV and this may explain the higher rate of anxiety in the first several months after diagnosis (20). This finding is in line with that of another study that found high rates of depression in relation to duration of infection (6), suggesting that the provision of mental health services at the time of diagnosis could be critical to manage the risk of depression and anxiety among recently-diagnosed individuals. Positive-affect skills interventions to support recently-diagnosed individuals have been shown to improve psychological health, decrease the use of anti-depressants, and support adjustment to HIV-positive status (33) and could be piloted in Chinese patient populations.

Consistent with other studies, we found the patients who reported high levels of support from family or friends had lower rates of symptoms of anxiety and depression (10, 34). This suggests that interventions designed to build the capacity of family and friends to support newly-diagnosed patients in the acceptance of their disease and in developing positive coping strategies could have mental health benefits.

Despite a high prevalence of anxiety and depression among HIV-infected persons, less than 5% of patients with borderline or likely depression were regularly using anti-depressants. Prior studies have also noted that most patients in China with anxiety or depression remain untreated and often have a poor understanding of available treatment options (20, 35). This is perhaps not surprising given the chronic and severe undersupply of mental health services in China (36, 37).

We did not find a relationship between high likelihood of depression and anxiety and clinical factors like immune function. However frequent switching of ART regimens was associated with higher proportion of depression, which makes sense: someone who has had trouble achieving or maintaining viral suppression is likely to experience anxiety and depression related to anticipated downward progression of their disease. Unlike in other studies (11, 19), patients on EFV-based regimens did not have elevated odds of anxiety or depression. However, this is likely due to a bias in the sample in that patients who had suffered CNS side effects like anxiety/depression from EFV may have switched regimens prior to this cross-sectional study, leaving only patients who did not suffer EFV-related side effects in the sample.

Asking patients to report experience of anxiety or depression in the previous month resulted in significant under-reporting of depression when compared to the HAD scales. Low recognition of mental health needs have been well studied in the US and Western Europe (8) and leads to under-utilization of mental health services. Fewer studies have been conducted in China, though two recent studies among rural residents reported low recognition of depression and anxiety (38) and low awareness of where to access mental health services (39). While introducing an initial screening question could be one way to identify a proportion of people who self-identify as experiencing mental health issues and these could be referred to mental health services, the low recognition of depression and anxiety in the population suggests that relying on such a question alone would miss a large proportion of patients who are identified as at risk of depression and anxiety through the HAD screening instrument. The introduction of a standardized assessment of mental health into HIV services could be done at hospitals where patients access ART or through the Center for Disease Control system that manages follow up and could be an effective way to identify patients who could benefit from clinical evaluation given that these mental health issues are treatable.

Our study had several potential limitations. The cross-sectional study design did not allow for assessment of the temporal relationship between anxiety/depression and factors (e.g., depression improvement over time or pre- and post- ART), the direction of causality, or assessment of whether anxiety/depression were transient or chronic in nature. Among our study's strengths is its large size and geographic diversity across 14 provinces in China and inclusion of many sub-populations. Also important was its use of validated measures to assess risk for anxiety and depression and that could easily be integrated into routine clinical practice (9, 22, 40).

In summary, the high prevalence of anxiety and depression symptoms among treatment-experienced HIV-infected individuals suggests that routinely administering a standardized mental health assessment to newly-diagnosed individuals and perhaps students would be a useful first step to identify those in need of further clinical evaluation. In order for the introduction of such an assessment to translate to better mental health outcomes, HIV clinics would need to identify optimal models for the provision of mental health interventions to their patients, whether through on-site service provision or a strong referral system to mental health professionals and other social support services.

## Author contributions

XH, HC, KM, and XinL led the analysis and writing of this manuscript. XH, KM, HaW, YC, KY, HC, and JH contributed to the final version. HaW is the Principle Investigator of and designed the study. XiaL, TZ, WX, HH, CL, SH, WC, HZ, CH, SL, HuW, XuL, PM, RY, GX, TL, DD, HC, YC, AS were involved in managing the data collection. All authors reviewed and approved the final version of the manuscript.

### Conflict of interest statement

The authors declare that the research was conducted in the absence of any commercial or financial relationships that could be construed as a potential conflict of interest.
